# Occupational human papillomavirus exposure risks for laryngeal surgeons and phoniatricians: A joint European Laryngological Society (ELS) and Union of European Phoniatricians (UEP) Delphi clinical consensus statement

**DOI:** 10.1007/s00405-026-10451-1

**Published:** 2026-07-19

**Authors:** Shiying Hey, Alberto Maria Saibene, Milan Amin, Jennifer Anderson, Christoph Arens, Simon Best, Guillermo Campos, Craig Derkay, Gauthier Desuter, Frederik G. Dikkers, Isabel Garcia-Lopez, Ahmed Geneid, Anastasios Hantzakos, Markus Hess, Yakubu Karagama, Marina Mat Baki, Nupur Kapoor Nerurkar, Daniel Novakovic, Haldun Oguz, Shazia Peer, Jasmine Prasad, Carlo Robotti, David Vokes, Chi-Te Wang, Małgorzata Wierzbicka, Michal Zabrodsky, Nicolien van der Poel

**Affiliations:** 1 Department of Otolaryngology, Guy’s and St Thomas’ NHS Foundation Trust, London, UK; 2 Otolaryngology Department, Department of Health Sciences, Santi Paolo e Carlo Hospital, Università degli Studi di Milano, Milan, Italy; 3 Otolaryngology-Head & Neck Surgery, NYU Grossman School of Medicine, New York, NY USA; 4 Department of Otolaryngology-Head & Neck Surgery, University of Toronto, Toronto, Canada; 5 Department of Otorhinolaryngology, Head & Neck Surgery, Plastic Surgery Gießen and Marburg University Hospitals, Justus-Liebig- University Gießen, Campus Gießen, Gießen, Germany; 6 Department of Otolaryngology-Head and Neck Surgery, Johns Hopkins University School of Medicine, Baltimore, MD USA; 7 Instituto de Laringología, Bogotá, DC Colombia; 8 Departments of Otolaryngology and Pediatrics, Eastern Virginia Medical School, Children’s Hospital of the King’s Daughters, Norfolk, VA USA; 9 Department of Otorhinolaryngology-Head & Neck Surgery, Antwerp University Hospital, Edegem, Belgium; 10 Department of Translational Neuroscience, Faculty of Medicine and Health Science, University of Antwerp, Antwerp, Belgium; 11 Department of Otorhinolaryngology/Head and Neck Surgery, Amsterdam UMC location Vrije Universiteit Amsterdam, De Boelelaan 1117, Amsterdam, The Netherlands; 12 Departamento de Otorrinolaringología y Cirugía de Cabeza y Cuello, Sección de Patología Vocal, Hospital Universitario La Paz, Madrid, Spain; 13 Department of Otorhinolaryngology and Phoniatrics-Head and Neck Surgery, Helsinki University Hospital, Helsinki, Finland; 14 Faculty of Medicine, University of Helsinki, Helsinki, Finland; 15 Department of Otolaryngology – Head & Neck Surgery, Integrated Subspecialties Institute, Cleveland Clinic Abu Dhabi, P.O. Box 112412, Abu Dhabi, United Arab Emirates; 16 Medical Voice Center, Hamburg, Germany; 17 King’s College Medical School, London, UK; 18 Department of Otorhinolaryngology—Head and Neck Surgery, Faculty of Medicine, Universiti Kebangsaan Malaysia, Kuala Lumpur, Malaysia; 19 Department of Otorhinolaryngology—Head and Neck Surgery, Hospital Canselor Tuanku Muhriz, Bandar Tun Razak, Cheras, Kuala Lumpur, Malaysia; 20 Bombay Hospital Voice and Swallowing Centre and ENT Department, Bombay Hospital and Medical Research Centre, Mumbai, Maharashtra India; 21 Voice Research Laboratory, Faculty of Medicine and Health, University of Sydney, Camperdown, NSW 2050 Australia; 22 Department of Otolaryngology, The Canterbury Hospital, Campsie, NSW 2194 Australia; 23 Fonomer Phoniatrics & Audiology Clinic, Ankara, Turkey; 24 Faculty of Medicine, Lokman Hekim University, Ankara, Turkey; 25 Division of Otorhinolaryngology, Head and Neck Surgery, Groote Schuur & Red Cross Children’s Hospitals, & The University of Cape Town, Cape Town, South Africa; 26 Otorhinolaryngology Department, San Giuseppe Hospital, IRCCS Multimedica, Milan, Italy; 27 Department of Otorhinolaryngology-Head and Neck Surgery, Auckland City Hospital, Auckland, New Zealand; 28 Department of Otolaryngology Head and Neck Surgery, Far Eastern Memorial Hospital, New Taipei City, Taiwan; 29 Department of Otolaryngology, Regional Specialist Hospital Wrocław, Research and Development Centre, Wrocław, Poland; 30 Faculty of Medicine, Wrocław University of Science and Technology, Wrocław, Poland; 31 Department of Otorhinolaryngology and Head and Neck Surgery, First Faculty of Medicine, University Hospital Motol, Charles University, Prague, Czech Republic

## Abstract

**Purpose:**

Laser surgical management of human papillomavirus (HPV)-related disease, such as recurrent respiratory papillomatosis (RRP), in the upper aerodigestive tract generates surgical smoke, which may represent a source of occupational viral exposure for laryngeal surgeons, phoniatricians, anesthesia providers and scrub nurses. Despite growing awareness, protective measures remain inconsistently implemented. This study aimed to develop an evidence-informed clinical consensus statement (CCS) to guide occupational HPV risk management in laryngology.

**Methods:**

A modified Delphi consensus process was conducted under the auspices of the European Laryngological Society (ELS) and the Union of the European Phoniatricians (UEP). A panel of 26 international experts undertook three rounds of voting on 18 statements across five domains: definitions and risk perception, personal protective measures, smoke evacuation and ventilation, preventive vaccination, and areas for further research.

**Results:**

All panellists completed all three rounds. Of the 18 statements, one reached strong consensus, seven reached consensus, one reached near consensus and six did not reach consensus. Three statements were merged across rounds. Key areas of consensus included the recognition of surgical smoke as a potential occupational HPV exposure risk, the inadequacy of standard surgical masks, the importance of offering HPV vaccination to healthcare workers with potential occupational exposure, alongside areas for further research.

**Conclusion:**

This joint ELS and UEP CCS represents the first internationally coordinated effort to address occupational HPV exposure in laryngology. While consensus was reached in several key areas, the findings underscore the need for targeted research and greater consistency in protective measures, educational provision and regulatory frameworks.

**Supplementary Information:**

The online version contains supplementary material available at https://doi.org/10.1007/s00405-026-10451-1.

## Introduction

Human papillomavirus (HPV) is a double-stranded DNA virus and is among the most prevalent sexually transmitted infections worldwide. Whilst high-risk subtypes such as HPV 16 and 18 are associated with malignant lesions of the cervix, oropharynx, and anogenital regions, low-risk HPV subtypes are typically manifested as benign verrucous proliferations. The most clinically significant of these within otolaryngological practice is recurrent respiratory papillomatosis (RRP), a benign epithelial neoplasm most commonly attributable to HPV subtypes 6 and 11, characterised by the development of exophytic papilloma arising from the squamous epithelium of the respiratory tract, with a marked predilection for the larynx. Despite its benign histopathology, RRP typically follows a chronic and relapsing course necessitating repeated surgical interventions across a patient’s lifetime [1]. 

The surgical management of both malignant and benign HPV-associated lesions, including laser ablation, laser excision, and electrosurgical excision, generates surgical smoke (SS), also referred to as surgical plume. SS comprises extremely small particles containing a complex mixture of chemical compounds and biological material, including viral DNA. Emerging evidence suggests that viable HPV particles may be present in SS, thereby raising concerns regarding potential occupational viral exposure among healthcare professionals [2]. The burden of HPV-related disease in otorhinolaryngology therefore extends beyond its direct clinical sequelae to encompass a potential and under-appreciated occupational hazard for the surgical team [2]. 

Despite growing awareness of the occupational hazards associated with SS and potential exposure to viral DNA, protective measures against occupational HPV exposure remain inconsistently implemented within laryngology. Whilst HPV DNA has been identified in SS and transmission risk has been explored across other surgical specialties, the occupational risk in otorhinolaryngology remains insufficiently characterised. This concern is further compounded by an increasing shift towards laser ablation of RRP in office-based settings, where protective measures may be less standardised than in the operating theatre. A recent worldwide survey of laryngeal surgeons and phoniatricians demonstrated marked variability in risk perception, use of personal protective equipment, use of plume evacuation systems, and HPV vaccination uptake, reflecting a persistent gap between emerging evidence and routine clinical practice and the need for clearer guidance within ENT specialties [2–4]. 

In response to this unmet need, the European Laryngological Society (ELS) and the Union of the European Phoniatricians (UEP) initiated this Delphi consensus process. This clinical consensus statement (CCS) aims to provide clinicians, institutions, and policymakers with practical, evidence-informed guidance to enhance occupational safety, promote the consistent adoption of preventive measures, and reduce potential HPV-related risks amongst professionals caring for patients with laryngeal and other HPV-associated diseases.

## Methods

**Fig. 1:**
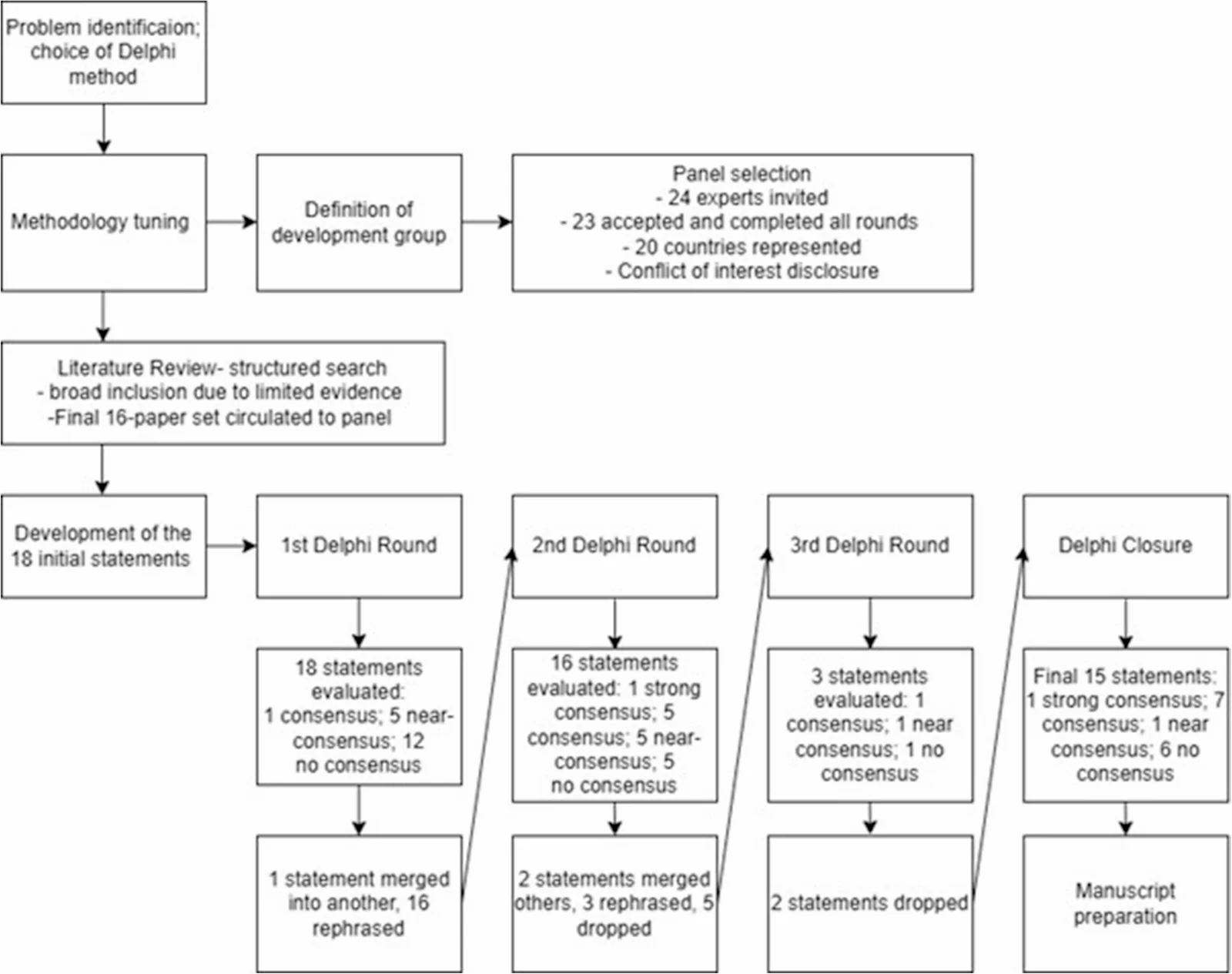
Workflow of the present CCS

The CCS was developed in accordance with the modified Delphi protocol proposed by Rosenfeld et al. and the Delphi standardised reporting recommendations (DELPHISTAR) [5, 6]. Building upon this fundamental methodological framework, the specific protocol was adapted from prior CCS developed by the same methodologist and further refined to minimise bias and improve clarity [7–10]. The Delphi survey was designed to undergo no more than three rounds, unless full agreement was reached during an earlier round (Fig. 1).

The modified Delphi method was selected given the limited and heterogeneous nature of the currently available evidence on occupational HPV exposure in laryngology, combined with the anticipated variability in expert opinion across an inherently controversial field that remains both insufficiently studied and regulated. In circumstances where the existing literature is insufficient to support formal evidence-based recommendations, the Delphi method offers a structured and transparent means of systematically eliciting and consolidating expert knowledge, while minimising the influence of individual bias. All methodological development was carried out exclusively by the development group, without external methodological consultation.

### CCS development group composition and panellists’ selection

The development group (DG) consisted of a chair (SH), an assistant chair (NP), and a methodologist (AMS). All DG members were board-certified otolaryngologists with expertise in adult and/or paediatric laryngology, laryngological clinical research and clinical consensus development methodology. The DG selected a panel of 24 otolaryngologists curated from different international societies: European Laryngology Society, (ELS), Union of the European Phoniatricians (UEP), British Laryngological Association (BLA) and American Laryngological Association (ALA). All experts were invited only when they had proven level of expertise based on clinical and/or scientific experience, with efforts made to maximise geographical distribution and gender diversity. Furthermore, panellists were selected on the basis of a variety of their skillset and expertise in office-based and operation-room based treatment of RRP. All potential panellists were contacted by email, with non-responders being sent two reminders before exclusion from the final panel. Prior to inclusion, all panellists were asked to disclose any existing or perceived competing interests relevant to the subject of the CCS.

### Scope of CCS

The CCS scope aims to enhance occupational safety, promote the consistent adoption of preventive measures, and ultimately reduce potential HPV-related risks amongst professionals caring for patients with laryngeal and other HPV-associated diseases, in both operating theatre and in-office settings. The target audience includes, but is not limited to, laryngeal surgeons and phoniatricians.

### Literature review

**Fig. 2:**
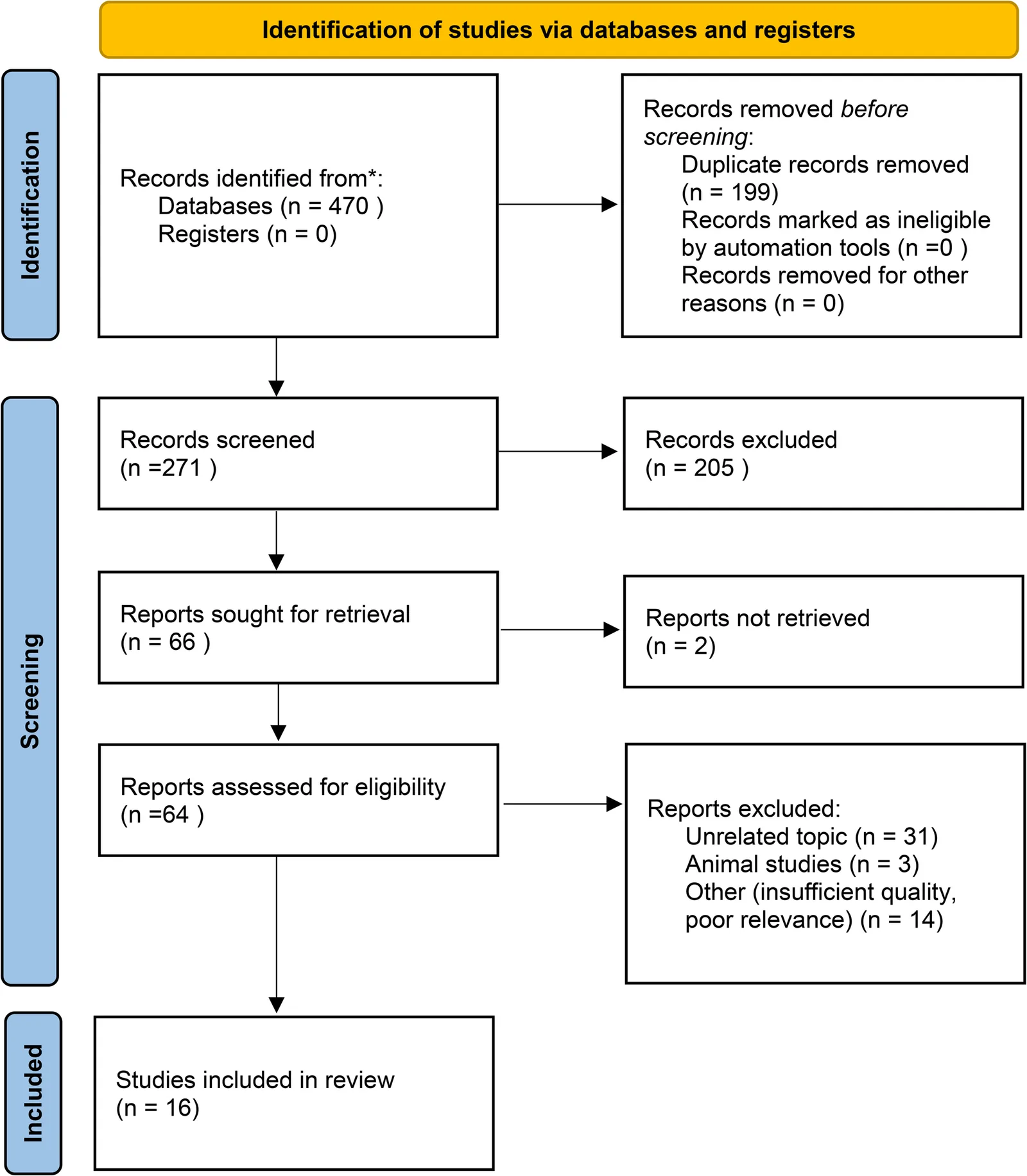
PRISMA flowchart detailing the study selection process

The DG, together with an independent researcher (JP), performed a scoping literature review across the MEDLINE database via PubMed, the Cochrane Library, the Web of Science Core Collection, and the EMBASE database via Ovid. The search query was: ‘HPV or “human papillomavirus” AND “occupational exposure"’. The reviewers had further utilised an ascendancy approach, screening the reference lists of identified sources for further eligible articles for inclusion. The search was conducted on 7th October 2024. In keeping with the CCS manual recommendations of Rosenfeld et al. [6], and given the limited literature available on this subject, the search was deliberately broad, encompassing all pertinent documents regardless of article format or study design. The DG reviewed all retrieved items and included or excluded them on the basis of relevance and overall quality, with any disagreements resolved by majority. Articles were considered for inclusion if published in English, Italian, Dutch, German, French, Chinese, or Spanish. The literature search yielded 271 unique results after removal of duplicates. Following title and abstract screening, 205 studies were excluded. Sixty-six articles underwent full-text review. After full text screening of 64 papers, 16 were selected for the review. Citations for all included studies are available in Supporting Information. The PRISMA flowchart detailing the study selection process is shown in Fig. 2. The final circulated collection comprised 16 articles (Table 1) which, in departure from conventional Delphi methodology, extended beyond reviews, systematic reviews and meta-analyses. The collection further encompassed case reports, non-human studies, and original research, reflecting the paucity of high-level evidence currently available on this subject. Panellists were given a month to review the literature and were invited to propose additional articles for circulation. At the end of the literature review phase, no further articles were submitted.

**Table 1:** Summary of findings from literature review

Author (Year)	Study Design	Specialty/Setting	Sample Size/Data Source	Summary
Systematic Reviews				
Palma et al. (2021)	Systematic review and meta-analysis	Surgical specialties performing ablation procedures on HPV-associated upper airway diseases	30 studies	HPV particles are dispersed in surgical smoke generated by CO2 laser and LEEP. Ablation procedure can lead to workplace contamination. CO2 laser users have a higher risk for warts of the upper airways compared to controls. While high-risk HPV transmission by surgical smoke is feasible, infection risk remains controversial in humans
Fox-Lewis et al. (2020)	Systematic review	Surgical procedures of HPV-related diseases with smoke generation and/or contamination	21 studies	HPV DNA has been identified in surgical smoke and contaminate upper airway of HCWs however, direct infectivity in humans remains uncertain. Safest to treat surgical smoke as potentially infectious. Precautionary measures including smoke evacuation, general room ventilation and fit-tested particulate respirator of at least N95 grade are recommended.
Robertson-Moore & Wu (2021)	Systematic review	Various surgical settings	35 studies	Definitive evidence shows that transmission of viable viral particles through SS is possible. Although several viral pathogens have a theoretical risk of transmission, only HPV has been shown to produce clinical infection in humans exposed to SS.
Cox et al. (2020)	Systematic review	Laser treatment of HPV-infected lesions	Multiple clinical and laboratory studies	HPV DNA has consistently been detected in laser plume with case reports suggest possible occupational transmission. Protective strategies and vaccination consideration are recommended.
Experimental Study				
Best et al. (2011)	Experimental animal study	Laboratory based	Murine papillomavirus infection model	In an experimental model of murine papillomavirus infections treated with surgical devices, the virus remains infectious; both the surgically treated tissue and the smoke plume is capable of transmitting infection.
Clinical Observational Study				
Ilmarinen et al. (2012)	Prospective observational study	ENT and dermatologic procedures	10 patients, 18 healthcare workers	HPV DNA identical to patient tissue was detected on surgical gloves but not on masks or oral mucosa of staff, suggesting contamination of protective equipment rather than direct transmission when PPE is used.
Survey Studies				
Afsar et al. (2024)	Cross-sectional survey	Healthcare providers performing HPV-related procedures	348 healthcare providers	Awareness of occupational HPV exposure exists but vaccination uptake remains low. Many eligible clinicians remain unvaccinated.
Stanley et al. (2018)	Cross-sectional survey	Gynaecologists and otolaryngologists	63 physicians	Most respondents had not been vaccinated against HPV, yet have largely favourable attitudes towards HPV vaccination and would consider vaccination in the future.
Williams et al. (2021)	Cross-sectional survey	Dermatologic surgeons	147 surgeons	Over half of the respondents had not received HPV vaccination. The overwhelming majority of respondents (89%) would be willing to become vaccinated if further evidence of human HPV transmission through surgical smoke emerged.
Chen et al. (2023)	International cross-sectional survey	Laryngeal surgeons	158 surgeons from 53 countries	Over half of respondents expressed concern about the risk of HPV transmission. Adaptation of safety protocols and personal protection varied widely and the majority were not vaccinated.
Narrative Reviews/Commentaries				
Harrison & Huh (2020)	Narrative review/commentary	Healthcare workers exposed to surgical plume	Literature synthesis	Evidence suggests potential occupational exposure to HPV via SS and authors advocate precautionary vaccination and improved protective measures.
Rypka et al. (2022)	Policy commentary	Dermatology practice	Literature discussion	Recommends routine HPV vaccination for dermatologists and healthcare professionals performing SS-generating procedures.
Bourgeois & Ross (2019)	Commentary	Dermatologic surgery	Narrative review	Highlights occupational exposure risk during wart ablation procedures and advocates vaccination for dermatologic surgeons.
Case Reports				
Hallmo & Naess (1991)	Case report	Laser surgery	1 surgeon	Development of laryngeal papillomatosis in a laser surgeon exposed to HPV plume suggested possible occupational transmission.
Rioux et al. (2013)	Case reports	Gynaecological laser surgery	2 surgeons	Two laser surgeons developed HPV-positive tonsillar carcinoma following long-term plume exposure.
Parker et al. (2019)	Case reports	Gynaecological electrosurgery	2 gynaecologists	HPV-positive oropharyngeal cancer reported in surgeons with prolonged electrosurgical plume exposure without consistent smoke evacuation.

### Clinical statement development

The DG compiled an initial list of 18 statements based on the literature review and these were subdivided into the following five sections: Definitions and Perception of Occupational HPV Risk, Personal Protective Measures, Smoke Evacuation and Ventilation Systems, Preventive Vaccination of Healthcare Workers, and Areas for Further Research. The draft statements were circulated among all panellists during the one-month literature review period, allowing sufficient time to propose additional statements prior to the voting phase. No new statement suggestions were received at the end of this period. Throughout the entire Delphi process, until voting for all rounds was completed, all panellists were contacted independently and individually to preserve blinding. Upon the commencement of the voting period, all panellists were contacted personally by email, and the survey comprising 18 statements was distributed via Google Forms (Google LLC, Mountain View, CA, USA). Panellists were instructed to complete the survey anonymously, facilitated by a randomly generated personal identification number provided by an independent collaborator (JP), allowing the DG to monitor response completion while preserving anonymity.

Each panellist was asked to indicate their level of agreement with each statement using a nine-point Likert scale (1 = strongly disagree; 9 = strongly agree), with the option to provide free text comments after each item. In accordance with the Rosenfeld et al. development manual [6], the following definitions were applied:

- Strong consensus: mean score of ≥ 8.00 with no outliers (defined as any rating two or more Likert points from the mean in either direction).
- Consensus: mean score of ≥ 7.00 with no more than one outlier.
- Near consensus: mean score of ≥ 6.50 with no more than two outliers.
- No consensus: all other statements.

All panellists were encouraged throughout the Delphi process to provide open comments, identify additional subtopics or statements and actively contribute to the debate, whether nominally or anonymously. All numerical results and open comments were shared anonymously with all panellists between rounds.

Statements not reaching consensus after the first round were rephrased or merged where perceived redundancy existed. After the second round, only statements demonstrating score improvement from the first round, namely those entering the non-consensus to near-consensus area, were carried forward for further refinement. When statements were rephrased or merged for a next round, comments of the panellists were analysed and used to refine the statements using the expert’s opinions and to gain a potential higher level of consensus in the next round. Non-improved items were dropped from the CCS. After the third round, all remaining statements not reaching consensus were dropped from the CCS. The three Delphi rounds were conducted one month apart to allow panellists adequate time for consideration and critical appraisal of the statements. Given the homogeneity of the panel, no subgroup analysis was anticipated.

### Article preparation and authorship

The original article draft was prepared by the DG with the assistance of an independent researcher (JP). The article was subsequently finalised by the DG, JP, and all panellists. Authorship was granted to the whole panel, the DG and JP. The chair, methodologist, and vice-chair of the DG were assigned first, second, and last author positions respectively, while all remaining authors were listed alphabetically as co-authors.

## Results

**Fig. 3:**
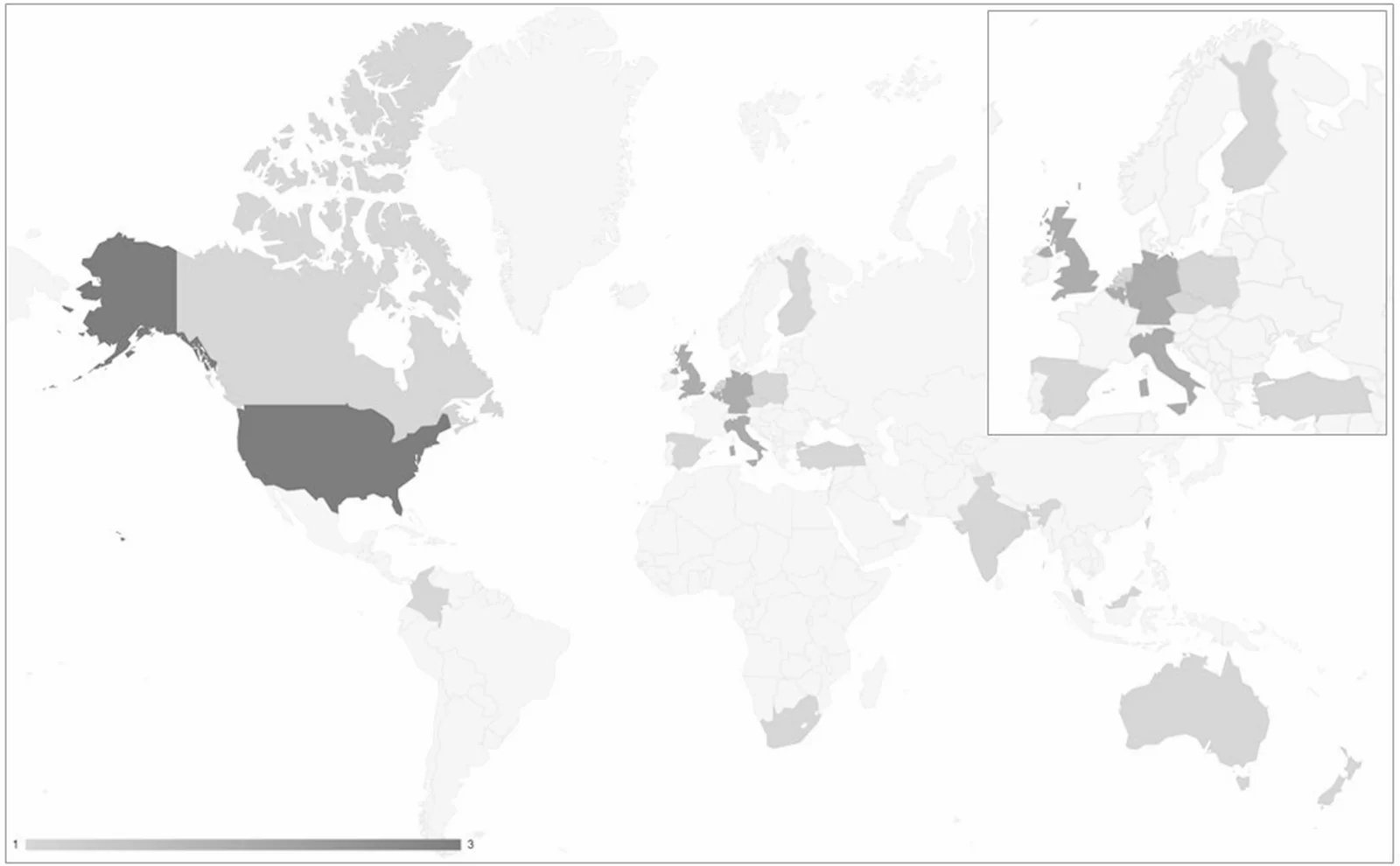
Geographic distribution of the panellists

The consensus process commenced in May 2025 and concluded in January 2026. The final panel comprised the DG and 23 additional international experts (out of 24 invited), collectively representing 20 countries worldwide (Fig. 3). None reported substantial or perceived conflicts of interest or bias towards the subject of this CCS. Excluding the DG, only the United States and Germany were represented by more than one panellist. All panellists participated in all three Delphi rounds.

Of the 18 statements presented in the first Delphi round, one reached consensus and five reached near consensus.

For the second round, the statement list was reduced to 16, following the carry forward of the one statement that had already reached consensus and the merging of two statements. Of these 16 statements, one reached strong consensus, five reached consensus and five reached near consensus. Three statements reaching near consensus in the second round were rephrased for the third and final round, whilst two further near-consensus statements were merged. In accordance with the study design, statements (5 out of 16) that did not demonstrate score improvement after two rounds were dropped from further voting.

In the third round, 3 statements were circulated, and among these, one statement reached consensus, one reached near consensus, and one did not reach consensus.

Across all three Delphi rounds, of the original 18 statements, one reached strong consensus, seven reached consensus, one reached near consensus, and six did not reach consensus. The remaining three statements were merged into other statements. Table 2 presents the final statements alongside their respective scores and consensus status. Supplementary Material 2a reports all statements, scores across each Delphi round, open comments, and the evolution of statements throughout the three rounds.

**Table 2:** Summary of Clinical Consensus Statement Outcomes

Section	Statement	Mean	Median	Range	Outliers	Result	Decision
I. Definitions and Perception of Occupational HPV Risk	***1. Surgical procedures for HPV-related upper aerodigestive tract lesions can generate surgical smoke containing HPV DNA***,*** which may represent an occupational exposure risk.***	8.19	9	3–9	1	CONSENSUS	Accepted in CCS
	***2 (merged with 3). Surgical procedures for HPV-related pathology in the upper aerodigestive tract***,*** such as recurrent respiratory papillomatosis***,*** may expose surgeons to aerosolised HPV particles. While viral material has been detected in surgical smoke***,*** the infectivity and viability of these particles remain uncertain.***	7.88	8	1–9	1	CONSENSUS	Accepted in CCS
	***4. Current educational programmes on occupational HPV risk for laryngeal surgeons are insufficient and require improvement***,*** ideally guided by consensus and stronger evidence.***	8.58	9	7–9	0	STRONG CONSENSUS	Accepted in CCS
II. Personal Protective Measures	***5. Standard three-ply surgical masks provide inadequate protection against aerosolised HPV in surgical smoke; higher-filtration***,*** well-fitted masks (e.g.***,*** N95) are recommended.***	7.92	8	4–9	1	CONSENSUS	Accepted in CCS
	***6. Fit-tested FFP3 (or equivalent) respirators are strongly recommended for HPV-related surgical procedures in the upper aerodigestive tract***,*** and should be made available and offered to healthcare workers.***	7.27	8	1–9	6	NO CONSENSUS	Dropped from CCS
	***7. Appropriate eye protection should be considered during HPV-related procedures to reduce mucosal exposure***,*** with the choice tailored to the surgical technique (e.g.***,*** microscope***,*** laser use) and environment (office-based vs. operating theatre).***	7.42	8	3–9	5	NO CONSENSUS	Dropped from CCS
	***8. Institutional PPE policies should explicitly address protection against HPV exposure in relevant surgical settings***,*** similar to policies for other high-risk aerosol-generating diseases (e.g.***,*** COVID-19***,*** pulmonary tuberculosis) and include information on vaccination for healthcare workers.***	7.46	8	2–9	3	NO CONSENSUS	Dropped from CCS
III. Smoke Evacuation and Ventilation Systems	***9. Efficient smoke evacuation or suction at source level should be routinely employed during HPV-related surgical procedures that generate smoke (e.g.***,*** during laser use)***,*** whether performed in office-based or operating theatre settings.***	7.96	9	2–9	2	NEAR CONSENSUS	Dropped from CCS
	***10 (merged with 11). HPV-related upper aerodigestive tract surgery should be performed in settings (operating room or office-based environment) with adequate general ventilation and appropriate air-exchange rates.***	8.04	8	5–9	4	NO CONSENSUS	Dropped from CCS
IV. Preventive Vaccination of Healthcare Workers	***12. HPV vaccination should be offered to healthcare workers***,*** including laryngeal surgeons***,*** who perform HPV-related procedures and are exposed to surgical smoke. While prior HPV exposure may have occurred***,*** the favourable safety profile and potential high benefit support vaccination in this group.***	8.58	9	5–9	1	CONSENSUS	Accepted in CCS
	***13 (merged with 14). HPV vaccination for healthcare workers should be accessible***,*** with institutional support where feasible***,*** for those who choose vaccination to mitigate potential occupational risk.***	8.38	9	5–9	3	NO CONSENSUS	Dropped from CCS
V. Areas for Further Research	***15. Additional studies are needed to quantify and compare HPV DNA presence and viral load in smoke plumes produced by different laser modalities (CO₂***,*** blue***,*** KTP) and devices.***	8.5	9	5–9	1	CONSENSUS	Accepted in CCS
	***16. Further research will be valuable to clarify the incidence of HPV infection or seroconversion among healthcare workers following occupational exposure***,*** taking into account prior HPV exposure and vaccination status.***	8.62	9	6–9	1	CONSENSUS	Accepted in CCS
	***17. Long-term cohort studies are needed to assess malignancy risk in surgeons with repeated occupational exposure to HPV-containing surgical smoke***,*** particularly from procedures involving high-risk HPV types.***	8.15	9	5–9	3	NO CONSENSUS	Dropped from CCS
	***18. Further research is warranted to clarify the role of vaccination in preventing occupationally acquired HPV infections in healthcare workers***,*** although demonstrating a direct causal link can present challenges.***	8.5	9	5–9	1	CONSENSUS	Accepted in CCS

The highest mean score (8.62) was recorded for Statement 16 (*Further research will be valuable to clarify the incidence of HPV infection or seroconversion among healthcare workers following occupational exposure*,* taking into account prior HPV exposure and vaccination status*). The strongest level of agreement, with no outliers, was achieved by Statement 4 (*Current educational programs on occupational HPV risk for laryngeal surgeons are insufficient and require improvement*,* ideally guided by consensus and stronger evidence*). Conversely, the lowest-scoring statement (mean 7.27) and the most divisive, attracting six outliers, was Statement 6 (*Fit-tested FFP3 or equivalent respirators are strongly recommended for HPV-related surgical procedures in the upper aerodigestive tract and should be made available and offered to healthcare workers*).).

## Discussion

This CCS was developed to raise awareness and provide practical, evidence-informed recommendations to mitigate potential HPV-related occupational exposure risks for laryngeal surgeons and phoniatricians.

### Definitions and perception of occupational HPV risk

All three statements in this section were accepted into the CCS. The panel reached consensus that surgical procedures for HPV-related upper aerodigestive tract lesions can generate surgical smoke containing HPV DNA, representing a potential occupational exposure risk. Equally, consensus was achieved on the recognition that while viral material has been detected in surgical smoke, the infectivity and viability of aerosolised HPV particles remain uncertain. Notably, the strongest level of agreement across the entire CCS was reached for Statement 4, reflecting unanimous recognition among panellists that current educational programmes on occupational HPV risk for laryngeal surgeons are insufficient and require improvement. These outcomes demonstrated that awareness of occupational HPV exposure risk is broadly shared. However, uncertainty remains on its clinical magnitude, underscoring the need for improved education and targeted research to address this gap.

### Personal protective measures (PPM)

The expert panel reached consensus only on the inadequacy of standard three-ply surgical masks for protection against aerosolised HPV in surgical smoke, reflecting broad agreement that higher-filtration, well-fitted masks offer superior respiratory protection. This aligns with growing experimental and epidemiological evidence demonstrating the presence of viral DNA in surgical plume and the limited filtration efficiency of conventional surgical masks for fine aerosols [11, 12]. 

In contrast, no consensus was reached for recommendations regarding fit-tested FFP3 respirators, routine eye protection, or explicit institutional PPE policies addressing HPV exposure. The lack of agreement likely reflects variability in perceived risk, differences in local resources and infrastructure and the absence of high-quality evidence directly linking specific PPE measures to mitigate HPV transmission risks in laryngology practice [13]. Additionally, heterogeneity in surgical techniques, procedural settings and national regulatory frameworks may have further influenced expert opinion. Thus far, the Association of peri-Operative Registered Nurses (AORN) has been a major advocate for the recognition of surgical smoke as an occupational health hazard and has actively lobbied for regulations in the United States. However, outside the United States, formal guidelines and regulatory frameworks addressing surgical smoke safety and personal protective measures remain limited or absent [13, 14]. 

### Smoke evacuation and ventilation systems

Of the two statements evaluated in this section, neither achieved full consensus. The statement on routine employment of efficient smoke evacuation or suction at source level during HPV-related surgical procedures reached only near consensus after three rounds and was therefore dropped from the final CCS. Similarly, consensus was not reached on the requirement for adequate general ventilation and appropriate air-exchange rates when HPV-related upper aerodigestive tract surgery is performed and this statement was discontinued after the second round.

Although discussion among panellists was generally supportive of the underlying principles, no clear consensus was reached. This was largely attributable to the inconclusive evidence base for their specific clinical application, as well as variation in the availability of dedicated smoke evacuation and ventilation systems across different settings. Concerns were also raised about the potential for jet ventilation to increase aerosol dispersion. In situations where smoke evacuation or specialised ventilatory systems are not available, the use of efficient suction positioned as close as possible to the smoke source, together with appropriate PPE, could be considered as the minimum standard.

Panel commentary further highlighted the distinction between operating theatre and office-based environments. While post-COVID infrastructure has to some extent addressed ventilation standards in operating theatre settings, office-based environments remain considerably less regulated. The increasing migration of RRP management to office-based settings, therefore, poses an occupational exposure burden that remains insufficiently quantified, and whether the smoke volume generated in such settings confers a comparatively lesser risk is uncertain. The compatibility of office-based ventilation with theatre-equivalent standards should not be assumed. Furthermore, whether additional measures such as HEPA filtration are warranted in office settings remains subject to individual surgeon risk assessment. Adequate suction and appropriate personal protection should nonetheless be considered in both settings.

### Preventive vaccination of healthcare workers

Vaccination of healthcare workers (HCWs) against HPV represented one of the most debated areas of the CCS. While all three statements achieved relatively high mean scores from the first round onward, none reached consensus initially due to marked dispersion of votes, highlighting persistent uncertainty rather than outright disagreement.

The statement advocating that HPV vaccination should be offered to HCWs with potential occupational exposure eventually reached consensus after rephrasing, suggesting that the panel converged on a position favouring access and availability rather than obligation. This evolvement mirrors the circulated literature evidence, which consistently documents the presence of HPV DNA in surgical smoke and, in experimental settings, evidence of infectivity [15]. This nonetheless stops short of providing definitive evidence of occupational transmission leading to clinical disease or seroconversion at a population level [11, 16]. 

Conversely, statements implying a stronger institutional or policy-driven responsibility for vaccination, or targeting specific “high-risk” subgroups of HCWs, failed to reach consensus and were eventually merged or discarded. This outcome reflects several considerations raised in panel commentary as well as in the literature, including the high background prevalence of HPV infection, heterogeneity in vaccination policies across countries, variable baseline immunity, and the absence of robust epidemiological data linking occupational exposure to incident HPV-related disease in HCWs. Various surveys amongst different surgical specialties further demonstrate wide variability in vaccination uptake and attitudes, often driven more by personal risk perception than by formal guidance [4, 17, 18]. 

Overall, the final consensus reflects a balanced position, endorsing vaccination as an available preventive measure while acknowledging that stronger recommendations cannot currently be justified solely on the basis of existing evidence.

### Areas for further research

Three of the four proposed statements on future research priorities reached consensus, underscoring the persistent uncertainty surrounding several aspects of occupational HPV exposure in laryngology. The panel agreed that further research is warranted to better quantify HPV DNA presence and viral load in surgical smoke, clarify the infectivity of aerosolised HPV, and evaluate the role of vaccination in preventing occupationally acquired HPV infections. Despite this, it was acknowledged that such investigations would be methodologically challenging, given the high background prevalence of HPV in the general population, multiple potential non-occupational transmission pathways and the widespread uptake of HPV vaccination among younger HCWs. Furthermore, these studies should be appropriately adopted based on the surgical modalities used (including but not limited to CO2, blue, KTP laser or cold steel techniques), as well as both office-based and operating theatre settings.

Nonetheless, carefully designed studies incorporating baseline HPV status, vaccination history and well-defined exposure cohorts may be feasible and would help advance the knowledge in our field.

Beyond specific research priorities, these findings highlight the broader importance and our aims of raising awareness among all healthcare workers potentially exposed to occupational HPV hazards, including but not limited to nursing staff, paramedics, trainees, students, etc. Vaccination coverage and healthcare regulations remain variable both between and within developed and developing countries, suggesting a potential role for future healthcare governance in promoting equitable access, targeted occupational health guidance and clearer recommendations regarding vaccination and preventive strategies for occupational HPV exposure in laryngology.

### Limitations

The low quality of currently available evidence represents a fundamental limitation of this CCS, given that the existing literature is largely derived from retrospective studies, case series, and case reports. Despite concerted efforts to assemble an internationally diverse panel, an inherent participation bias should also be acknowledged. While the panel comprised only specialist laryngologists and phoniatricians, it is anticipated that their perspectives would have relevance to a broader population of healthcare workers with occupational HPV exposure. However, it is acknowledged that these expert opinions may not be fully generalisable to other staff groups, including residents, fellows, and support personnel.

Some of the statements within the CCS had high means of accordance, but could not reach consensus due to the number of outliers, as we strictly adhered to Rosenfeldt’ guideline for CCS development. This continues to reflect the limited availability of high quality evidence on this topic, and the fact that some statements are highly dependent on local practices and resources, resulting in potential geographical variations.

The literature review was necessarily restricted to articles published in English, Italian, German, Chinese, Dutch, French, and Spanish, potentially excluding relevant studies published in other languages. Similarly, despite the panel’s international composition, regional and country-specific perspectives and practices could not be fully represented. Finally, while the modified Delphi method provides a structured and systematic framework for consensus building, it remains reflective of expert opinion shaped by local practices, resources and regulatory contexts.

## Conclusions

This joint ELS and UEP clinical consensus statement represents the first internationally coordinated effort to address occupational HPV exposure risks amongst laryngeal surgeons, phoniatricians and members of the operative team. While the panel reached consensus on several key areas, including the recognition of surgical smoke as a potential vector for HPV transmission, the inadequacy of current educational programmes and the importance of offering HPV vaccination to healthcare workers with potential occupational exposure, a number of statements did not reach consensus. This reflects not indifference to the issue, but the genuine paucity of high-quality evidence currently available to support more definitive recommendations. Until stronger evidence emerges, the use of adequate surgical smoke suction, appropriate PPE, and vaccination as protective measures across both operating room and office-based settings should be guided by individual risk assessment. Furthermore, as office-based laryngological practice continues to expand, active consideration of these issues will be essential to safeguarding the health of the surgical workforce.

The findings of this CCS continue to underline the need for targeted and methodologically robust research to better characterise the occupational burden of HPV exposure in laryngology, quantify viral load in surgical smoke and evaluate the protective role of vaccination in our population. As key stakeholders, clinicians, institutions and policymakers are encouraged to recognise occupational HPV exposure as a workplace health concern and to work towards greater consistency in protective measures, educational provision, and regulatory frameworks.

## Supplementary Information

Below is the link to the electronic supplementary material.

Supplementary Material 1

Supplementary Material 2

## References

[1] So RJ, McClellan K, Best SR (2023) Recurrent Respiratory Papillomatosis: Quality of Life Data from an International Patient Registry. Laryngoscope 133:1919–1926. 10.1002/lary.30401 36177852

[2] Georgesen C, Lipner SR (2018) Surgical smoke: Risk assessment and mitigation strategies. J Am Acad Dermatol 79:746–755. 10.1016/j.jaad.2018.06.003 29902546

[3] Ostapovych U, Vortman R (2022) Implementing a surgical smoke evacuation policy and procedure: a quality improvement project. AORN J 115:139–146. 10.1002/aorn.13603 35084765

[4] Chen T, Prasad J, Robotti C et al (2025) Human Papillomavirus Exposure Risk and Vaccination Awareness Among Laryngeal Surgeons: A Worldwide Survey Study. Laryngoscope 135:4303–4311. 10.1002/lary.32401 40613547

[5] Niederberger M, Schifano J, Deckert S et al (2024) Delphi studies in social and health sciences-recommendations for an interdisciplinary standardized reporting (DELPHISTAR). Results of a Delphi study. PLoS One 19:e0304651. 10.1371/journal.pone.0304651 PMC11346927 39186713

[6] Rosenfeld RM, Nnacheta LC, Corrigan MD (2015) Clinical Consensus Statement Development Manual. Otolaryngol Head Neck Surg 153:S1–S14. 10.1177/0194599815601394 26527615

[7] van der Poel N, Shah G, Wolter N et al (2025) Management of pott’s puffy tumor: a Delphi-method international clinical consensus statement. Eur Arch Otorhinolaryngol 282:4669–4680. 10.1007/s00405-025-09589-1 PMC12423165 40782155

[8] van der Poel N, Saibene AM, Simon F et al (2025) Postoperative management of pediatric tympanostomy tubes: a Yo-IFOS consensus. Eur Arch Otorhinolaryngol 282:5613–5622. 10.1007/s00405-025-09485-8 40579482

[9] Urbanelli A, Pugliese G, Bolis E et al (2025) Delphi Consensus in Otolaryngology: A Systematic Review of Reliability and Reporting Completeness. J Pers Med 15. 10.3390/jpm15120567 PMC12734207 41440930

[10] Saibene AM, Nitro L, Carsuzaa F et al (2025) Comprehensive management of cocaine-induced midline destructive lesions: a Young-IfOS consensus. J Pers Med. 10.3390/jpm15060231 PMC12193749 40559094

[11] Fox-Lewis A, Allum C, Vokes D, Roberts S (2020) Human papillomavirus and surgical smoke: a systematic review. Occup Environ Med 77:809–817. 10.1136/oemed-2019-106333 32385189

[12] Gao S, Koehler RH, Yermakov M, Grinshpun SA (2016) Performance of Facepiece Respirators and Surgical Masks Against Surgical Smoke: Simulated Workplace Protection Factor Study. Ann Occup Hyg 60:608–618. 10.1093/annhyg/mew006 PMC7109898 26929204

[13] Benaim EH, Jaspers I (2024) Surgical smoke and its components, effects, and mitigation: a contemporary review. Toxicol Sci 198:157–168. 10.1093/toxsci/kfae005 PMC10964745 38243717

[14] Spruce L (2021) Surgical Smoke Safety. AORN J 114:493–501. 10.1002/aorn.13543 34706079

[15] Best SR, Esquivel D, Mellinger-Pilgrim R et al (2020) Infectivity of murine papillomavirus in the surgical byproducts of treated tail warts. Laryngoscope 130:712–717. 10.1002/lary.28026 PMC6884657 31041820

[16] Robertson-More C, Wu T (2021) A knowledge gap unmasked: viral transmission in surgical smoke: a systematic review. Surg Endosc 35:2428–2439. 10.1007/s00464-020-08261-5 PMC7833447 33495880

[17] Afsar S, Hossain M, Islam M et al (2024) Human papillomavirus and occupational exposure: the need for vaccine provision for healthcare providers. Hum Vaccin Immunother. 10.1080/21645515.2024.2342622 PMC11110707 38771122

[18] Stanley C, Secter M, Chauvin S, Selk A (2018) HPV vaccination in male physicians: A survey of gynecologists and otolaryngology surgeons’ attitudes towards vaccination in themselves and their patients. Papillomavirus Res 5:89–95. 10.1016/j.pvr.2018.03.001 PMC5887017 29524677

