# Supplementary material for: Occupational human papillomavirus exposure risks for laryngeal surgeons and phoniatricians: A joint European Laryngological Society (ELS) and Union of European Phoniatricians (UEP) Delphi clinical consensus statement
Source: Eur Arch Otorhinolaryngol. 2026 Jul 19;283(10):6569–81. doi: 10.1007/s00405-026-10451-1 (PMC13614997; doi:10.1007/s00405-026-10451-1)
Supplement: Supplementary file 1 [file 405_2026_10451_MOESM2_ESM.docx]

| **Section** | **First round** | | | | | | | **Second round** | | | | | | | **Third round** |  |  |  |  |  |  |
| --- | --- | --- | --- | --- | --- | --- | --- | --- | --- | --- | --- | --- | --- | --- | --- | --- | --- | --- | --- | --- | --- |
|  | **Statement** | **Mean** | **Median** | **Range** | **Outliers** | **Result** | **Decision** | **Statement** | **Mean** | **Median** | **Range** | **Outliers** | **Result** | **Decision** | **Statement** | **Mean** | **Median** | **Range** | **Outliers** | **Results** | **Decision** |
| **I. Definitions and Perception of Occupational HPV Risk** | ***1. HPV-related upper aerodigestive tract surgical procedures can generate surgical smoke containing viable HPV DNA representing an occupational exposure risk*** | 8.15 | 9 | 4-9 | 3 | NO CONSENSUS | Edited for 2nd round | ***1. Surgical procedures for HPV-related upper aerodigestive tract lesions can generate surgical smoke containing HPV DNA, which may represent an occupational exposure risk.*** | 8.19 | 9 | 3-9 | 1 | CONSENSUS | Accepted in CCS |  |  |  |  |  |  |  |
|  | ***2. Patient-to-surgeon HPV transmission has been documented during HPV-related surgery, particularly when surgical smoke is produced.*** | 7.42 | 8 | 3-9 | 4 | NO CONSENSUS | Edited for 2nd round | ***While patient-to-surgeon HPV transmission has been reported during HPV-related surgery, particularly in the presence of surgical smoke, the evidence is limited and direct causality can be difficult to establish.*** | 7.58 | 8 | 2-9 | 2 | NEAR CONSENSUS | Edited for 3rd round | ***2. Surgical procedures for HPV-related pathology in the upper aerodigestive tract, such as recurrent respiratory papillomatosis, may expose surgeons to aerosolised HPV particles. While viral material has been detected in surgical smoke, the infectivity and viability of these particles remain uncertain.*** | 7.88 | 8 | 1-9 | 1 | CONSENSUS | Accepted in CCS |
|  | ***3. Surgical procedures for HPV-related pathology in the upper aerodigestive tract (e.g., recurrent respiratory papillomatosis) can expose operating surgeons to aerosolised HPV particles.*** | 8.19 | 9 | 5-9 | 2 | NEAR CONSENSUS | Edited for 2nd round | ***Surgical procedures for HPV-related pathology in the upper aerodigestive tract (e.g., recurrent respiratory papillomatosis) may expose surgeons to aerosolised HPV particles, though the viability of these particles remains uncertain.*** | 7.81 | 8 | 1-9 | 2 | NEAR CONSENSUS | Merged with statement 2 |  |  |  |  |  |  |  |
|  | ***4. Current educational programmes on occupational HPV risk for laryngeal surgeons are insufficient and requires improvement.*** | 8.04 | 9 | 3-9 | 2 | NEAR CONSENSUS |  | ***Current educational programmes on occupational HPV risk for laryngeal surgeons are insufficient and require improvement, ideally guided by consensus and stronger evidence.*** | 8.58 | 9 | 7-9 | 0 | STRONG CONSENSUS | Accepted in CCS |  |  |  |  |  |  |  |
| **II. Personal Protective Measures** | ***5. Standard three-ply surgical masks do not provide adequate protection against aerosolised HPV in surgical smoke.*** | 7.54 | 9 | 2-9 | 4 | NO CONSENSUS | Edited for 2nd round | ***Standard three-ply surgical masks provide inadequate protection against aerosolised HPV in surgical smoke; higher-filtration, well-fitted masks (e.g., N95) are recommended.*** | 7.92 | 8 | 4-9 | 1 | CONSENSUS | Accepted in CCS |  |  |  |  |  |  |  |
|  | ***6. Fit-tested FFP3 (or equivalent) respirators should be mandatory for all HPV-related surgical procedures in the upper aerodigestive tract, including surgery for recurrent respiratory papillomatosis.*** | 7.31 | 8 | 2-9 | 5 | NO CONSENSUS | Edited for 2nd round | ***Fit-tested FFP3 (or equivalent) respirators are strongly recommended for HPV-related surgical procedures in the upper aerodigestive tract, and should be made available and offered to healthcare workers.*** | 7.27 | 8 | 1-9 | 6 | NO CONSENSUS | Dropped from CCS |  |  |  |  |  |  |  |
|  | ***7. Eye protection (including laser-rated goggles or plastic face shields) should be worn routinely during HPV-related procedures to reduce mucosal exposure.*** | 6.77 | 7 | 2-9 | 10 | NO CONSENSUS | Edited for 2nd round | ***Appropriate eye protection should be considered during HPV-related procedures to reduce mucosal exposure, with the choice tailored to the surgical technique (e.g., microscope, laser use) and environment (office-based vs operating theatre).*** | 7.42 | 8 | 3-9 | 5 | NO CONSENSUS | Dropped from CCS |  |  |  |  |  |  |  |
|  | ***8. Institutional PPE policies should explicitly address protection against HPV exposure in relevant surgical settings.*** | 8.19 | 9 | 5-9 | 3 | NO CONSENSUS | Edited for 2nd round | ***Institutional PPE policies should explicitly address protection against HPV exposure in relevant surgical settings, similiar to policies for other high-risk aerosol-generating diseases (e.g.,COVID-19, pulmonary tuberculosis) and include information on vaccination for healthcare workers.*** | 7.46 | 8 | 2-9 | 3 | NO CONSENSUS | Dropped from CCS |  |  |  |  |  |  |  |
| **III. Smoke Evacuation and Ventilation Systems** | ***9. Local smoke evacuation systems with ULPA or HEPA filters should be used during all HPV-related surgical procedures.*** | 8 | 8.5 | 5-9 | 3 | NO CONSENSUS | Edited for 2nd round | ***Local smoke evacuation systems with ULPA or HEPA filtration should be available and used during HPV-related surgical procedures, particularly when smoke is generated (e.g., during laser use).*** | 8.27 | 9 | 5-9 | 2 | NEAR CONSENSUS | Edited for 3rd round | ***9. Efficient smoke evacuation or suction at source level should be routinely employed during HPV-related surgical procedures that generate smoke (e.g., during laser use), whether performed in office-based or theatre settings.*** | 7.96 | 9 | 2-9 | 2 | near consensus | Dropped from CCS |
|  | ***10. Operating rooms used for HPV-related upper aerodigestive tract surgery should provide adequate general ventilation with appropriate air-exchange rates.*** | 8.46 | 9 | 5-9 | 2 | NEAR CONSENSUS | Edited for 2nd round | ***HPV-related upper aerodigestive tract surgery should be performed in settings (operating room or office-based environment) with adequate general ventilation and appropriate air-exchange rates.*** | 8.04 | 8 | 5-9 | 4 | NO CONSENSUS | Dropped from CCS |  |  |  |  |  |  |  |
|  | ***11. Smoke-evacuation equipment should be available and used for office-based procedures treating HPV-related upper aerodigestive tract disease (e.g., recurrent respiratory papillomatosis).*** | 7.5 | 9 | 2-9 | 6 | NO CONSENSUS | Merged with statement 10 |  |  |  |  |  |  |  |  |  |  |  |  |  |  |
| **IV. Preventive Vaccination of Healthcare Workers** | ***12. HPV vaccination should be offered to healthcare workers, including laryngeal surgeons, who perform HPV-related procedures and are exposed to surgical smoke.*** | 8.23 | 9 | 2-9 | 3 | NO CONSENSUS | Edited for 2nd round | ***HPV vaccination should be offered to healthcare workers, including laryngeal surgeons, who perform HPV-related procedures and are exposed to surgical smoke. While prior HPV exposure may have occurred, the favourable safety profile and potential high benefit support vaccination in this group.*** | 8.58 | 9 | 5-9 | 1 | CONSENSUS | Accepted in CCS |  |  |  |  |  |  |  |
|  | ***13. Healthcare institutions should facilitate and subsidise HPV vaccination for employees at occupational risk of HPV exposure.*** | 8.35 | 9 | 5-9 | 3 | NO CONSENSUS | Edited for 2nd round | ***Healthcare institutions should consider facilitating and subsidising HPV vaccination for employees at occupational risk of HPV exposure, unless previously vaccinated, similar to established practices for other occupational infection risks (e.g., hepatitis B).*** | 8.34 | 9 | 5-9 | 2 | NEAR CONSENSUS | Edited for 3rd round | ***13. HPV vaccination for healthcare workers should be accessible, with institutional support where feasible, for those who choose vaccination to mitigate potential occupational risk.*** | 8.38 | 9 | 5-9 | 3 | no consensus | Dropped from CCS |
|  | ***14. HPV vaccination for at-risk healthcare workers should be recommended irrespective of age or gender.*** | 8.12 | 9 | 4-9 | 4 | NO CONSENSUS | Edited for 2nd round | ***HPV vaccination should be considered for all at-risk healthcare workers, although regional or national regulations and age-based criteria may pose challenges to implementation.*** | 8.35 | 9 | 3-9 | 2 | NEAR CONSENSUS | Merged with statement 13 |  |  |  |  |  |  |  |
| **V. Areas for Further Research** | ***15. Additional studies are needed to quantify and compare HPV DNA presence and viral load in smoke plumes prodcuced by different laser modalities (CO₂, blue, KTP) and devices.*** | 8.5 | 9 | 5-9 | 1 | CONSENSUS | Accepted in CCS |  |  |  |  |  |  |  |  |  |  |  |  |  |  |
|  | ***16. The incidence of HPV infection or seroconversion among healthcare workers after occupational exposure requires further investigation.*** | 8.35 | 9 | 5-9 | 2 | NEAR CONSENSUS | Edited for 2nd round | ***Further research will be valuable to clarify the incidence of HPV infection or seroconversion among healthcare workers following occupational exposure, taking into account prior HPV exposure and vaccination status.*** | 8.62 | 9 | 6-9 | 1 | CONSENSUS | Accepted in CCS |  |  |  |  |  |  |  |
|  | ***17. Long-term cohort studies are needed to assess malignancy risk in surgeons with repeated occupational exposure to HPV-containing surgical smoke.*** | 7.89 | 9 | 3-9 | 3 | NO CONSENSUS | Edited for 2nd round | ***Long-term cohort studies are needed to assess malignancy risk in surgeons with repeated occupational exposure to HPV-containing surgical smoke, particularly from procedures involving high-risk HPV types.*** | 8.15 | 9 | 5-9 | 3 | NO CONSENSUS | Dropped from CCS |  |  |  |  |  |  |  |
|  | ***18. Further research is needed to evaluate the effectiveness of HPV vaccination in preventing occupationally acquired infections in healthcare workers.*** | 8.38 | 9 | 6-9 | 2 | NEAR CONSENSUS | Edited for 2nd round | ***Further research is warranted to clarify the role of vaccination in preventing occupationally acquired HPV infections in healthcare workers, although demonstrating a direct causal link can present challenges.*** | 8.5 | 9 | 5-9 | 1 | CONSENSUS | Accepted in CCS |  |  |  |  |  |  |  |
